# Schistosome and liver fluke derived catechol-estrogens and helminth associated cancers

**DOI:** 10.3389/fgene.2014.00444

**Published:** 2014-12-23

**Authors:** José M. Correia da Costa, Nuno Vale, Maria J. Gouveia, Mónica C. Botelho, Banchob Sripa, Lúcio L. Santos, Júlio H. Santos, Gabriel Rinaldi, Paul J. Brindley

**Affiliations:** ^1^Center for Parasite Biology and Immunology, National Health Institute Doutor Ricardo JorgePorto, Portugal; ^2^Center for the Study of Animal Science, Instituto de Ciências e Tecnologias Agrárias e Agroalimentares, University of PortoPorto, Portugal; ^3^Department of Chemistry and Biochemistry, Centro de Investigação em Química, University of PortoPorto, Portugal; ^4^Department of Health Promotion and Chronic Diseases, National Health Institute Doutor Ricardo JorgePorto, Portugal; ^5^Tropical Disease Research Laboratory, Liver Fluke and Cholangiocarcinoma Research Center, Department of Pathology, Faculty of Medicine, Khon Kaen UniversityKhon Kaen, Thailand; ^6^Experimental Pathology and Therapeutics Group, Portuguese Institute for Oncology of PortoPorto, Portugal; ^7^Research Center for Neglected Diseases of Poverty, Department of Microbiology, Immunology and Tropical Medicine, School of Medicine & Health Sciences, George Washington UniversityWashington, DC, USA

**Keywords:** urogenital schistosomiasis, opisthorchiasis, catechol-estrogens, oxysterols, DNA-adducts, neglected tropical disease-associated-cancer, squamous cell carcinoma of the bladder, cholangiocarcinoma

## Abstract

Infection with helminth parasites remains a persistent public health problem in developing countries. Three of these pathogens, the liver flukes *Clonorchis sinensis*, *Opisthorchis viverrini* and the blood fluke *Schistosoma haematobium,* are of particular concern due to their classification as Group 1 carcinogens: infection with these worms is carcinogenic. Using liquid chromatography-mass spectrometry (LC-MS/MS) approaches, we identified steroid hormone like (e.g., oxysterol-like, catechol estrogen quinone-like, etc.) metabolites and related DNA-adducts, apparently of parasite origin, in developmental stages including eggs of *S. haematobium*, in urine of people with urogenital schistosomiasis, and in the adult stage of *O. viverrini*. Since these kinds of sterol derivatives are metabolized to active quinones that can modify DNA, which in other contexts can lead to breast and other cancers, helminth parasite associated sterols might induce tumor-like phenotypes in the target cells susceptible to helminth parasite associated cancers, i.e., urothelial cells of the bladder in the case of urogenital schistosomiasis and the bile duct epithelia or cholangiocytes, in the case of *O. viverrini* and *C. sinensis*. Indeed we postulate that helminth induced cancers originate from parasite estrogen-host epithelial/urothelial cell chromosomal DNA adducts, and here we review recent findings that support this conjecture.

## BIOLOGICAL CARCINOGENS – THREE HELMINTH PARASITES

**FIGURE 1:**
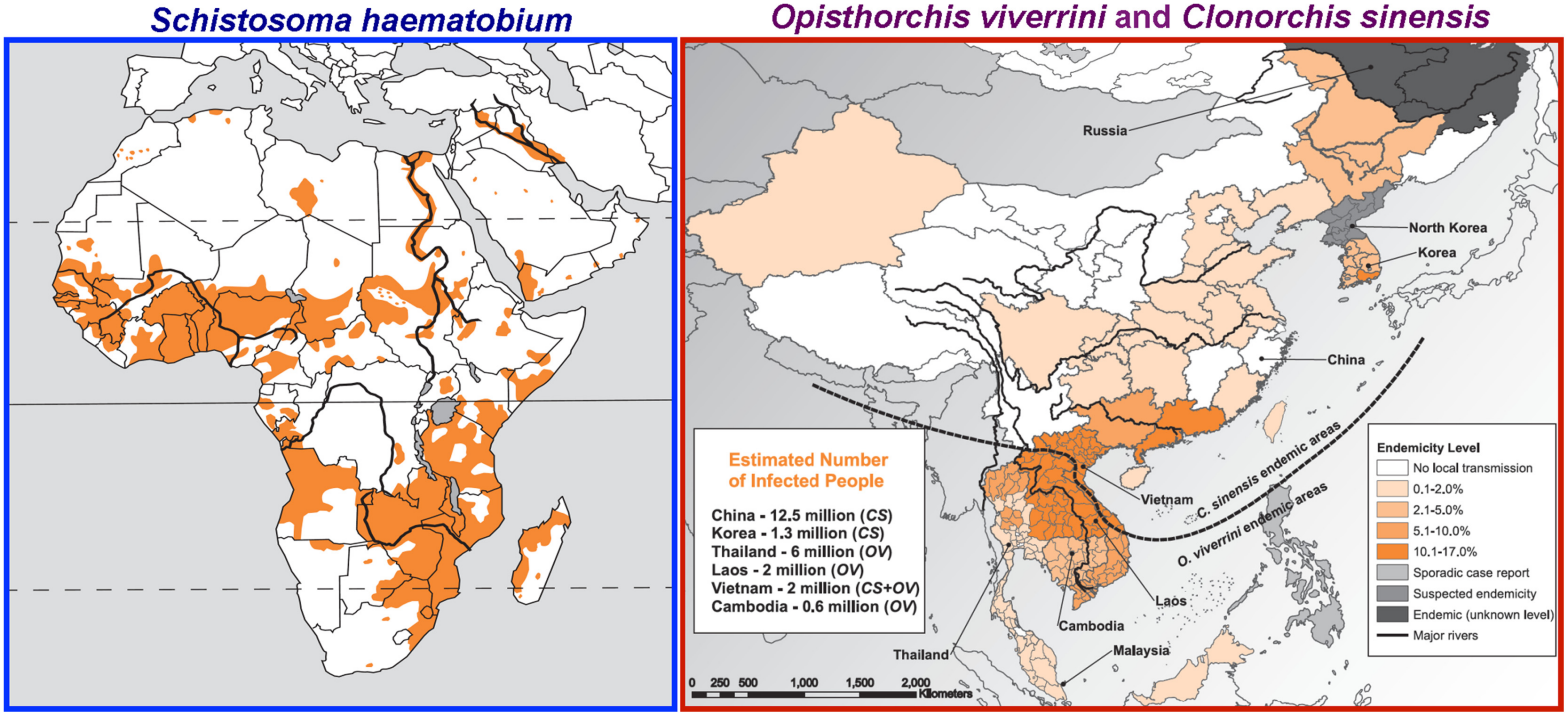
**Geographical distribution of the carcinogenic flukes.** Left: Distribution of the blood fluke *Schistosoma haematobium* (sub-Saharan Africa, Nile valley in Egypt and Sudan, the Maghreb, and the Arabian peninsula). Right: Distribution of the liver flukes *Clonorchis sinensis* (China, Republic of Korea, Democratic People’s Republic of Korea, the Russian Federation, and northern Viet Nam) and *Opisthorchis viverrini* (Thailand, Laos, Cambodia, Malaysia, and southern part of Viet Nam). “No local transmission” stands for “no reported local transmission.” From International Agency for Research on Cancer (IARC), (2012); permission requested from WHO Press, International Agency for Research on Cancer.

The World Health Organization’s International Agency for Research on Cancer (IARC) and the United States’ National Institutes of Health (NIH) consider that ~20% of cancers are caused by infectious diseases. Some cancer-inducing infectious agents, such as Hepatitis B and C Viruses, are well known. However, less appreciated are the several major human helminth pathogens that cause cancer. IARC recognizes three helminth infections as definitive causes of cancer – the fish-borne liver flukes *Opisthorchis viverrini* and *Clonorchis sinensis* and the blood fluke *Schistosoma haematobium* (Bouvard et al., 2009; de Martel et al., 2012; International Agency for Research on Cancer (IARC), 2012; **Figure 1**). In addition to direct detriment on development and health of infected populations, infection with liver flukes and schistosomes – types of helminth parasites collectively termed trematode flatworms – lead to infection related cancers, specifically cholangiocarcinoma (CCA; bile duct cancer) and squamous cell carcinoma (SSC) of the urinary bladder, respectively.

## UROGENITAL SCHISTOSOMIASIS AND BLADDER CANCER

Three major species of schistosomes are the agents of human schistosomiasis – *Schistosoma japonicum* and *Schistosoma mansoni* cause intestinal schistosomiasis in East Asia, Africa, South America, and the Caribbean while *S. haematobium*, occurring widely through Africa and the Middle East, causes urogenital schistosomiasis (**Figure 1**). In the range of 4.5–70 million disability adjusted life years (DALYs) are lost to schistosomiasis (King and Dangerfield-Cha, 2008). More people are infected with *S. haematobium* than with the other schistosomes. Of ~112 million cases of *S. haematobium* infection in sub-Saharan Africa, 70 million are associated with hematuria, 18 million with major bladder wall pathology, and 10 million with hydronephrosis leading to kidney damage (van der Werf et al., 2003; Hotez et al., 2009; King, 2010). In many patients, deposition of *S. haematobium* parasite ova eventually leads to SSC of the bladder (Hodder et al., 2000; Parkin, 2006). Moreover, as many as 75% of women infected with *S. haematobium* suffer from female genital schistosomiasis (FGS) of the lower genital tract (Hotez et al., 2009). FGS results from deposition of schistosome eggs in the uterus, cervix, vagina and vulva, with ensuing inflammatory responses. It impairs fertility (Santos et al., 2014) and also increases susceptibility of the woman to HIV (Feldmeier et al., 1994; Kjetland et al., 2006; Ndhlovu et al., 2007; Jourdan et al., 2011).

Squamous cell carcinoma is a malignant, poorly differentiated neuroendocrine neoplasm. SCC is the common form of bladder cancer in rural Africa where *S. haematobium* is prevalent (Mostafa et al., 1999; Zhong et al., 2013). In contrast, the majority of bladder cancer in developing countries and regions not endemic for urogenital schistosomiasis is transitional cell carcinoma (TCC) that arises from the transitional epithelium lining of the bladder. The parasite eggs trapped in the bladder wall release antigens and other metabolites (presumably evolved to expedite egress to the urine, and hence to the external environment). Nonetheless, the phenomenon leads to hematuria and to chronic inflammation, in turn increasing risk of urothelial hyperplasia, dysplasia, and SCC of the bladder (Honeycutt et al., 2014). The epidemiologic association between SSC of the bladder with schistosomiasis haematobia is based both on case control studies and on the correlation of bladder cancer incidence with prevalence of infection with *S. haematobium* within different geographic locations. Schistosomiasis haematobia is a chronic infection, the adult, egg-producing schistosomes live for many years, re-infections frequently occur, and schistosomiasis associated bladder SCC appears relatively early, often by the mid-decades of life. By contrast, TCC usually presents in the later decades of life. The incidence of urogenital schistosomiasis associated SCC is estimated in 3–4 cases per 100,000 (Shiff et al., 2006).

## FISH-BORNE FLUKES AND BILE DUCT CANCER

Liver infection caused by *O. viverrini*, *C. sinensis* and related flukes remains a major public health problem in East Asia and Eastern Europe where >40 million people are infected. *O. viverrini* is endemic in Thailand, Lao PDR, Vietnam and Cambodia (Sripa et al., 2011; Sithithaworn et al., 2012; **Figure 1**). Humans acquire the infection with *O. viverrini* by eating undercooked, fresh water cyprinoid fish infected with the metacercariae of the fluke (Sripa et al., 2011). There the parasites mature over 6 weeks into adult flukes, which graze on biliary epithelia. Eggs of *O. viverrini* are shed in bile and exit the infected person with the fecal stream. Freshwater snails ingest the eggs; the parasite (and related flukes, above) undergoes transformations within the snail host, culminating in the release of cercariae that seek out and penetrate the skin of a freshwater fish. Where sanitation is less than optimal, eggs may enter fresh water ecosystems where the eggs are ingested by freshwater snails. Human infection leads to hepatobiliary disease, cholangitis, obstructive jaundice, hepatomegaly, periductal fibrosis, cholecystitis, and cholelithiasis (Blechacz et al., 2011; Mairiang et al., 2012). More problematically, experimental and epidemiological evidence implicates liver fluke in the etiology of a major sub-type of liver cancer, CCA or bile duct cancer [Bouvard et al., 2009; de Martel et al., 2012; International Agency for Research on Cancer (IARC), 2012].

Cholangiocarcinoma, bile duct cancer, is an adenocarcinoma of the bile ducts, with a dismal prognosis. These are slow growing tumors, which spread along bile ducts with periductal and mass forming extensions. Prognosis is poor owing to the silent clinical character, difficulty in early diagnosis, and limited therapeutic approaches, especially in resource poor settings such as northeastern Thailand where the recent estimate of median survival time after supportive treatment was 4 months (Thunyaharn et al., 2013). Surgical management is the only potentially curative treatment, but is restricted to early-stage disease. CCA has a worldwide distribution, beyond East Asia, where patients often develop CCA *de novo* without obvious risk factors. Primary sclerosing cholangitis and congenital bile duct anomalies are also precursors. In Thailand and elsewhere in East Asia, where infections with liver flukes are definitive risk factor, the factors share a common determinant of chronic inflammation and chronic injury of the biliary epithelium, including from persistent parasitism by these fish-borne trematodes (Sripa et al., 2009, 2012; Blechacz et al., 2011; Johnson et al., 2012; O’Hara et al., 2013; Razumilava and Gores, 2013).

## FLUKES, CATECHOL-ESTROGENS, OXYSTEROLS, AND CARCINOGENESIS

**FIGURE 2:**
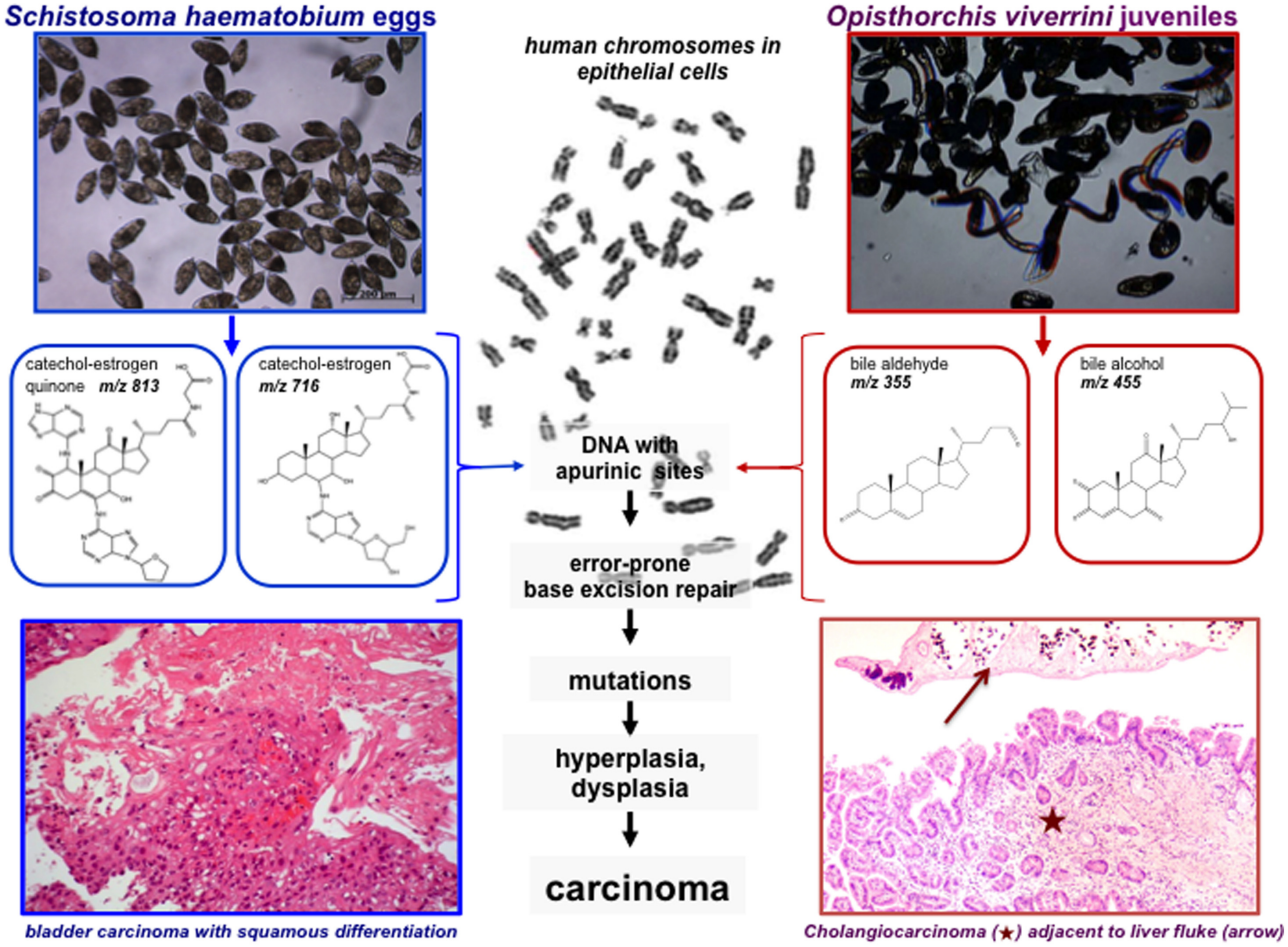
**Carcinogenesis mediated by steroid hormone like molecules derived from *S. haematobium* and *O. viverrini*.** Eggs of *S. haematobium* derived catechol-estrogens and DNA-adducts (left; Botelho et al., 2013), and *O. viverrini* derived oxysterols (right; Vale et al., 2013), likely interact with the chromosomes of target cells inducing DNA apurinic sites that eventually escape the DNA repair mechanisms leading to mutations. These mutations ultimately would transform the target cell, leading to hyperplasia and ultimately to neoplasia, i.e., squamous cell carcinoma of the bladder [bottom left, brief description adapted from Santos et al. (2014)] and liver fluke induced cholangiocarcinoma (CCA; bottom right). Hematoxylin and eosin stained section of human intrahepatic bile duct/liver, cholangiocarcinoma at bottom of image (star) and an adult *O. viverrini* liver fluke (arrow) at top. Image contributed by co-author Banchob Sripa. Human metaphase chromosomes – image from Tang et al. (2013).

In addition to the hormone-like effects of the parasite estradiol-related molecules on the endocrine and immune system of the host, initiation metabolites of estrogens can be also considered as carcinogenic chemicals (Cavalieri and Rogan, 2011, 2014). Hydroxylation of estrogens forms the 2- and 4-catechol estrogens involved in further oxidation to semiquinones and quinones, including formation of the catechol estrogen-3, 4-quinones, the major carcinogenic metabolites of estrogens. These electrophilic compounds react with macromolecules, including DNA, to form the depurinating adducts that eventually lead to mutations and cancer initiation (**Figure 2**; Cavalieri and Rogan, 2011). Several mechanisms explain the role of estrogens in disease. The better-known hypothesis is that the estrogen receptor mediates cell proliferation, increasing errors in DNA replication (Clemons and Goss, 2001; Yager and Davidson, 2006; Botelho et al., 2009b, 2013). Another interpretation postulates that estrogen metabolites react covalently with DNA bases by redox cycling or by forming an abasic site. Subsequent error-prone repair of the modified DNA generates oncogenic mutations that initiate cancer. The two mechanisms may act in concert. According to the second mechanism, P450 metabolism of estrone and estradiol generates the catechol estrogens, 2-hydroxyestrogen and 4-hydroxyestrogen. Further oxidation leads to 2, 3-catechol-estrogen quinine and 3, 4-catechol-estrogen quinone, respectively, which can react directly with DNA via a Michael addition or indirectly via generation of reactive oxygen species. Methylation of catechol estrogens by catechol-*O*-methyltransferase, conjugation of the catechol estrogen quinones with glutathione, and enzymatic reduction to reform catechol estrogens are processes that prevent accumulation of the highly reactive metabolites. However, if the latter protective processes are insufficient, catechol estrogen quinones accumulate, which damage DNA either by oxidation or depurination, and release of catechol estrogen modified purines (Liehr et al., 1986; Cavalieri et al., 1997; Yager and Davidson, 2006).

While examining human cases of urogenital schistosomiasis from Angola, we observed elevation in levels of estradiol in sera but not luteinizing hormone (LH) or testosterone (Botelho et al., 2009a). Estradiol is a steroid hormone secreted principally by the ovarian follicles in vertebrates. It seemed implausible that the elevated levels could be attributed to hypothalamic–pituitary–gonadal axis regulation. Rather, we speculated that schistosomes produced the estradiol-related metabolites that contributed to the elevated estradiol levels. Using mass spectrometry approaches (Gouveia et al., 2013) we characterized >20 estradiol related metabolites, from sera of *S. haematobium*-infected persons from Angola and, remarkably, in the parasites including the eggs (Botelho et al., 2013). Catechol-estrogens are formed by hydroxylation on the steroid aromatic ring A. Hydroxylation of both C-2 and C-3 on a steroid ring was apparent and, further, oxidation to an estradiol-2,3-quinone. The schistosome estrogenic metabolites readily seen in urine and *in vitro* appear to arise by reactions of quinones of catechol estrogens with chromosomal DNA (Botelho et al., 2011b, 2013). In addition, we exposed non-cancerous CHO (Chinese hamster ovary) cells to secretions and lysates of *S. haematobium eggs* and adult parasites, which stimulated cellular proliferation, migration and invasion, inhibited apoptosis, up-regulated expression of *Bcl-2*, and facilitated loss of *p27* in CHO (Botelho et al., 2009a,b, 2010, 2011a,b, 2013) – processes that are hallmarks of tumorigenesis and cancer cell survival (Hanahan and Weinberg, 2000). If similar phenomena also occur in human urogenital schistosomiasis, we speculate that they contribute to the abnormal proliferation and accumulation of genetic changes that occur in schistosomiasis-associated carcinogenesis [**Figure 2**; Mostafa et al., 1999; International Agency for Research on Cancer (IARC), 2012].

Opisthorchiasis is associated with elevation of bile acids, including deoxycholic acid (Vale et al., 2013) which are potent tumor promoters in cholangiocarcinogenesis (Sirica, 2005). Bile acids are synthesized in the liver from cholesterol, and the majorities are conjugated with either glycine or taurine (Haswell-Elkins et al., 1994; Ohshima et al., 1994; Akaike et al., 2003; Pinlaor et al., 2003; Katsuma et al., 2005; Sirica, 2005; Yongvanit et al., 2012). Inflammation-related carcinogenesis has also been associated to oxidative and nitrative DNA damage as 8-oxo-7,8-hydro-2′-deoxiguanine (8-oxodG) and 8-nitroguanine (8-NG; Yongvanit et al., 2012). Increased levels of nitrate and nitrite, which reflect endogenous generation of NO, occur during *O. viverrini* infection in humans (Haswell-Elkins et al., 1994) and rodents (Ohshima et al., 1994). Oxysterols, which are oxidation products of cholesterol generated by enzymatic (P450) or non-enzymatic processes (Jaworski et al., 2001; Jusakul et al., 2011), can be mutagenic or genotoxic, and to possess pro-oxidative and pro-inflammation properties that promote carcinogenesis. Investigation of binding domains in human genes has demonstrated an association between different types of oxysterols and the development and progression of cancer of the colon, lung, breast and bile ducts (Jaworski et al., 2001). Bile acids constitute a large family of steroids carrying a carboxyl group in the side chain. Bile alcohols have similar products in bile acid biosynthesis or as end products. We found these compounds in extracts of *O. viverrini* (**Figure 2** compound 18) but conjugated at different positions, free bile acids re-conjugated in some species like aldehydes (**Figure 2** compound 12) or as sulfates (not shown). The effects of these individual species can be anticipated to be structure-dependent, and metabolic conversions will result in a complex mixture of biologically active and inactive forms (Vale et al., 2013).

## INFECTION WITH BLOOD FLUKES AND LIVER FLUKES AS THE RISK FACTOR – BUT HOW MIGHT CANCER ARISE?

Current understanding of how infections with these flukes lead to cancers has been reviewed recently (Sithithaworn et al., 2012; Sripa et al., 2012; Honeycutt et al., 2014). In brief, in regions of high prevalence of opisthorchiasis, the risk factors for bile duct cancers are chronic inflammation and concomitant chronic injury of the biliary epithelium as the consequence of persistent parasitism by these fish-borne pathogens (Sripa et al., 2009, 2012; Blechacz et al., 2011; Johnson et al., 2012; O’Hara et al., 2013; Razumilava and Gores, 2013). The risk of SSC of the bladder during urogenital schistosomiasis appears to be promoted by concurrent risk factors associated with bladder cancer where infection with *S. haematobium* is less common or in non-endemic regions including exposure to toxins such as dyes from industrial and agricultural sources, and from tobacco smoke (see Honeycutt et al., 2014). Thus there are likely to be multiple factors including a diet rich in nitrosamines, spillover effects from local and systemic chronic inflammation (reactive oxygen species, reactive nitrogen species) directed against the worms, the secretion of mitogens and other mediators by the parasite (Satarug et al., 1998; Sripa et al., 2012), and interactions or changes in the biliary, GI tract and urinary tract microbiota, including co-infection by other potentially oncogenic biological species (Plieskatt et al., 2013).

To this list, we now include another potential mechanism: lesions in chromosomes and production of depurinating estrogen-DNA adducts leading to parasite metabolite-promoted host cell DNA damage, due to parasite-derived, reactive oxysterol and/or catechol estrogen derivatives. These processes contribute to urogenital schistosomiasis associated SCC during chronic urogenital schistosomiasis, and to CCA during chronic opisthorchiasis (**Figure 2**). Overall, the structures that we have identified in *S. haematobium* and *O. viverrini* (Botelho et al., 2013; Vale et al., 2013) suggest that carcinogenesis-related steroids may be released in carcinogenic quantities by these flukes. Notably, a relation between putative oxysterol or bile acid metabolites from *O. viverrini* and bile duct cancer has long been hypothesized (Changbumrung et al., 1990).

## CONCLUDING COMMENTS

Infection with helminth parasites remains a persistent public health problem in developing countries. Three of these pathogens, *C. sinensis*, *O. viverrini*, and *S. haematobium,* are of particular concern due to their classification by the IARC as Group 1 carcinogens. Infection with these worms is definitively associated with cancer. We have reported novel sterol-like metabolites and DNA-adducts in *S. haematobium*, in urine of persons with urogenital schistosomiasis, and in *O. viverrini*. Because these molecules are metabolized to active quinones that can modify DNA, helminth parasite associated catechol estrogens might induce tumor-like phenotypes in the epithelia of the bile ducts and bladder. Whereas the roles of these new metabolites in bile duct cancer and SSC of the bladder remain to be examined in depth, this clearly is worthy of deeper investigation. Future studies might profitably aim for isolation or chemical synthesis of these putative carcinogens and downstream investigation of interactions of the fluke estrogens and oxysterols with informative cells such as bladder urothelial cells (Botelho et al., 2013) and cholangiocytes (Grubman et al., 1994), and with oxysterol binding proteins and so forth. The interrelations of these carcinogens and the microbiota of the infected bladder and biliary system can also be predicted to be informative (Plieskatt et al., 2013). Moreover, given that other metabolites of *O. viverrini* are predicted to play a role in carcinogenesis of *O. viverrini* induced bile duct cancer, including liver fluke granulin (Smout et al., 2009), it will be informative also to compare and contrast action of liver fluke granulin and other fluke metabolites in these analyses, investigations that are now facilitated by the availability of genome sequences of these carcinogenic flukes (Wang et al., 2011; Young et al., 2012, 2014; Brindley and Hotez, 2013; Huang et al., 2013), genome sequences of CCAs (Chan-On et al., 2013), new rodent models (Fu et al., 2012), and functional genomic approaches developed for these parasites (Rinaldi et al., 2011, 2012). In addition to their carcinogenic effects, these flukes-associated sterol derivatives and DNA-adducts could be exploited as diagnostic and prognostic biomarkers, indeed 8-oxo dG in urine associates with opisthorchiasis-induced CCA (Thanan et al., 2008), and as targets for novel intervention strategies against these neglected tropical disease-associated cancers.

## Conflict of Interest Statement

The authors declare that the research was conducted in the absence of any commercial or financial relationships that could be construed as a potential conflict of interest.
